# Cladribine Combined with Low-Dose Cytarabine as Frontline Treatment for Unfit Elderly Acute Myeloid Leukemia Patients: Results from a Prospective Multicenter Study of Polish Adult Leukemia Group (PALG)

**DOI:** 10.3390/cancers13164189

**Published:** 2021-08-20

**Authors:** Bożena Katarzyna Budziszewska, Aleksander Salomon-Perzyński, Katarzyna Pruszczyk, Joanna Barankiewicz, Agnieszka Pluta, Grzegorz Helbig, Anna Janowska, Marta Kuydowicz, Łukasz Bołkun, Jarosław Piszcz, Elżbieta Patkowska, Marzena Wątek, Piotr Małecki, Sylwia Kościołek-Zgódka, Edyta Cichocka, Grzegorz Charliński, Anna Irga-Staniukiewicz, Jan Maciej Zaucha, Agnieszka Piekarska, Tomasz Gromek, Marek Hus, Karol Wójcik, Małgorzata Raźny, Mariola Sędzimirska, Bartosz Puła, Sebastian Giebel, Sebastian Grosicki, Agnieszka Wierzbowska, Ewa Lech-Marańda

**Affiliations:** 1 Department of Hematology, Institute of Hematology and Transfusion Medicine, 02-776 Warsaw, Poland; asalomon@ihit.waw.pl (A.S.-P.); kpruszczyk@ihit.waw.pl (K.P.); jbarankiewicz@ihit.waw.pl (J.B.); epatkowska@ihit.waw.pl (E.P.); mwatek@ihit.waw.pl (M.W.); bpula@ihit.waw.pl (B.P.); ewamaranda@ihit.waw.pl (E.L.-M.); 2 Centre of Postgraduate Medical Education, 01-813 Warsaw, Poland; 3 Department of Hematology, Medical University of Lodz, 93-513 Lodz, Poland; agnieszka.pluta@op.pl (A.P.); sekretariat.hemat@kopernik.lodz.pl (A.J.); mkuydowicz@su.krakow.pl (M.K.); agnieszka.wierzbowska@umed.lodz.pl (A.W.); 4 Department of Hematology and Bone Marrow Transplantation, School of Medicine in Katowice, Medical University of Silesia, 40-032 Katowice, Poland; ghelbig@o2.pl; 5 Department of Hematology, Medical University of Bialystok, 15-276 Bialystok, Poland; lukasz.bolkun@umb.edu.pl (Ł.B.); jaroslaw.piszcz@umb.edu.pl (J.P.); 6 Department of Hematology and Cancer Prevention, Faculty of Health Sciences, Medical University of Silesia, 40-055 Katowice, Poland; hematologia@zsm.com.pl (P.M.); sgrosicki@wp.pl (S.G.); 7 Center of Oncology, Hematology Department, 25-734 Kielce, Poland; Sylwia.Kosciolek-Zgodka@onkol.kielce.pl; 8 Hematology Department, Nicolaus Copernicus Hospital, 87-100 Torun, Poland; edytacichocka@gmail.com; 9 Warmian-Masurian Cancer Center of The Ministry of The Interior and Administration’s Hospital, Department of Hematology, 10-228 Olsztyn, Poland; grzegorz.charlinski@poliklinika.net; 10 Department of Hematology and Transplantology, Medical University of Gdansk, 80-952 Gdansk, Poland; airga@uck.gda.pl (A.I.-S.); jan.zaucha@gumed.edu.pl (J.M.Z.); babajaga@gumed.edu.pl (A.P.); 11 Department of Hematooncology and Bone Marrow Transplantation, Medical University of Lublin, 20-081 Lublin, Poland; tgromek@spsk1.lublin.pl (T.G.); markhus@o2.pl (M.H.); 12 Hematology Department, Rydygier Memorial Hospital, 31-826 Krakow, Poland; kwojcik@rydygierkrakow.pl (K.W.); m.razny@wp.pl (M.R.); 13 Lower Silesian Centrum for Cellular Transplantation, 53-439 Wroclaw, Poland; sedzimirska@dctk.wroc.pl; 14 Department of Bone Marrow Transplantation and Onco-Hematology, Maria Sklodowska-Curie National Research Institute of Oncology, Gliwice Branch, 44-102 Gliwice, Poland; Sebastian.Giebel@io.gliwice.pl

**Keywords:** acute myeloid leukemia, elderly, unfit patients, cladribine, low-dose cytarabine, non-intensive therapy

## Abstract

**Simple Summary:**

Treatment of acute myeloid leukemia (AML) in elderly patients unfit for intensive chemotherapy (IC) is a challenge in clinical practice. Here we prospectively evaluated a novel low-intensity regimen consisting of low-dose cytarabine combined with cladribine (LD-AC+cladribine) for remission induction followed by LD-AC alone in the maintenance phase as the frontline treatment for elderly AML patients ineligible for IC. We included a cohort of 117 elderly patients in poor performance status or with significant comorbidities. High-risk or intermediate-risk cytogenetics were observed in almost 90% of patients. Treatment with LD-AC+cladribine led to the objective response rate of 54% and the median overall survival of 17.3 months in the responders group. The toxicity profile was predictable and infectious complications were the most common non-hematological adverse events. In conclusion, we found LD-AC+cladribine as a beneficial therapeutic option with an acceptable safety profile in the difficult-to-treat population of elderly AML patient ineligible for IC.

**Abstract:**

Acute myeloid leukemia (AML) in older unfit patients is a therapeutic challenge for clinical hematologists. We evaluated the efficacy and safety of a novel low-intensity regimen consisting of low-dose cytarabine and cladribine (LD-AC+cladribine) in first-line treatment of elderly (≥60 years) AML patients not eligible for intensive chemotherapy (IC) who had either the Eastern Cooperative Oncology Group performance status (ECOG PS) ≥2 or the hematopoietic cell transplantation comorbidity index (HCT-CI) score ≥3. The induction phase included two cycles of LD-AC+cladribine. Patients who achieved at least partial remission (PR) received maintenance treatment with LD-AC alone. Overall, 117 patients with a median age of 70 years were enrolled. Adverse cytogenetics, ECOG PS ≥2 and HCT-CI score ≥3 was observed in 43.5%, 60%, and 58% of patients, respectively. The response rate (≥PR) was 54% (complete remission [CR], 32%; CR with incomplete hematologic recovery [CRi], 5%). A median overall survival (OS) was 21 and 8.8 months in CR/CRi and PR group, respectively. Advanced age (≥75 years) and adverse cytogenetics had a negative impact on OS. The 56-day mortality rate was 20.5%. In conclusion, LD-AC+cladribine is a beneficial therapeutic option with a predictable safety profile in elderly AML patients not eligible for IC.

## 1. Introduction

Acute myeloid leukemia (AML) most commonly affects older adults with a median age of 68 years at diagnosis [1]. The AML landscape changes with the increasing age of patients with high-risk cytogenetics, overexpression of genes contributing to treatment resistance, secondary AML after antecedent hematological disorders (sAML), and therapy-related AML (tAML), more frequently observed in older patients [2,3,4,5,6,7]. Simultaneously, due to age-related comorbidities and deterioration of general condition, the ability of elderly patients to tolerate intensive chemotherapy (IC) significantly decreases. Nevertheless, it is well known that chronological age alone is not a sufficient criterion to disqualify a patient from IC, and in some older adults, intensive treatment may result in a survival advantage [8,9,10]. Assessing whether a particular elderly patient will achieve benefits from IC is difficult and requires thorough clinical and, especially, geriatric evaluation [11,12]. Several predictive models are helpful in the decision-making process [13,14,15,16,17], the most commonly used being the hematopoietic cell transplantation comorbidity index (HCT-CI). In this model, a score of 2 or more defines a subgroup of older adults characterized by lower remission rate, increased early mortality, and decreased survival when IC is applied [13,14].

While any anti-leukemic therapy has been proven to be better than no therapy [18], as many as 60% of elderly AML patients do not receive any anti-leukemic treatment [19]. On the other hand, before the era of novel agents, those elderly patients who were offered treatment but were considered unfit for IC suffered from limited treatment options, including hypomethylating agents (HMA) (i.e., azacitidine [20,21,22,23] and decitabine [24,25,26]) and low-dose cytarabine (LD-AC) [20,24], which were generally unsatisfactory in terms of long-term disease control.

Cladribine, a purine analogue, exerts cytotoxic, proapoptotic, and antiproliferative effects on AML cells. Additionally, it inhibits DNA repair and acts as a hypomethylating and epigenetic agent [27]. Cladribine is able to act synergistically with other anti-leukemic drugs, in particular with AC (by increasing the intracellular concentration of the active AC metabolite in leukemic cells) and with anthracyclines [27,28]. Moreover, its cellular efflux is only minimally dependent on the P-glycoprotein, whose increased activity is known as one of the main mechanisms of drug resistance in AML [29]. As the activity of the P-glycoprotein increases with age, this feature of cladribine appears to be of particular importance in elderly AML patients [29].

The addition of cladribine to standard IC has improved the outcomes of younger AML patients in both frontline [30,31] and relapsed/refractory settings [32,33]. The randomized phase II trial confirmed benefit in overall survival (OS) from the incorporation of cladribine to daunorubicin and AC induction in the subgroup of treatment-naïve physically fit AML patients over the age of 60 with good or intermediate cytogenetics [34]. Recently, a novel combination of cladribine with granulocyte colony-stimulating factor, LD-AC, and aclarubicin has shown significant activity as salvage therapy for relapsed/refractory AML. As hematological toxicity was low, this regimen may be a suitable treatment option for elderly AML patients [35].

The prognosis of elderly AML patients remains poor with the 5-year OS rate of 5% [36]. To improve outcomes in this difficult-to-treat population of AML patients, we investigated a novel non-intensive therapeutic approach consisting of LD-AC in combination with cladribine (LD-AC+cladribine) for remission induction followed by LD-AC monotherapy in maintenance phase. Here we present the final results of a prospective multicenter study conducted by the Polish Adult Leukemia Group (PALG) assessing efficacy and safety of LD-AC+cladribine in the frontline treatment of elderly AML patients with poor performance status (PS) or a high burden of comorbidities.

## 2. Materials and Methods

### 2.1. Human Subjects Considerations

This prospective observational multicenter study conducted by PALG was approved by the Ethics Committee of the Institute of Hematology and Transfusion Medicine in Warsaw (No 31/2016). The study was conducted in accordance with the provisions of the 1964 Helsinki Declaration with its later amendments and the International Conference on Harmonization Guidelines for Good Clinical Practice. Written informed consent was obtained from all study participants.

Patients with newly diagnosed AML, according to the World Health Organization criteria [37], who were ≥60 years of age and had either the Eastern Cooperative Oncology Group (ECOG) PS of ≥2 or HCT-CI score of ≥3 were eligible for enrollment. Patients with tAML, AML with myelodysplasia-related changes (AML-MRC), and sAML were also included. Other active malignancy and concomitant uncontrolled infection were the main exclusion criteria. Prior treatment for the antecedent hematologic disorders was allowed.

### 2.2. Treatment Regimen

Induction therapy consisted of two cycles of cladribine 5 mg/m^2^ intravenously (IV) over 1–2 h on days 1 to 5 (in the first cycle) and on days 1 to 3 (in the second cycle) combined with LD-AC 40 mg subcutaneously (SQ) once daily on days 1–10 (in the first and second cycle). The first and second cycle of LD-AC+cladribine were administered with an interval of at least 4 weeks depending on blood count recovery and resolution of non-hematologic toxicities to grade 2 according to the National Cancer Institute Common Terminology Criteria for Adverse Events version 4.0 (CTCAE v. 4.0). Maintenance treatment with LD-AC 40 mg/m^2^ SQ on days 1 to 10 given every 4 to 6 weeks was offered to all patients who achieved at least a partial remission (PR) after a second cycle of LD-AC+cladribine. Patients continued to receive treatment until disease progression or unacceptable toxicity or withdrawal of consent. Patients who did not respond to 2 cycles of LD-AC+cladribine were offered the best supportive care or palliative treatment according to the physician’s decision.

### 2.3. Study Procedures

Bone marrow (BM) aspirations were performed at screening and after the second cycle of LD-AC+cladribine. Additional BM aspirations were obtained when disease progression was suspected. The investigators evaluated both treatment responses and cytogenetic risk according to the 2017 European LeukemiaNet guidelines [38]. Molecular testing was not mandatory and included screening for mutations in *FLT3* (both for internal tandem duplications [ITDs] and tyrosine kinase domain [TKD] mutations at codons D835 and I836), *NPM1*, *CEBPA*, and *RUNX1* genes.

Safety was evaluated by adverse events (AEs) assessment, medical history, physical examination, concomitant medications, and clinical laboratory tests. Investigator-assessed AEs were graded according to the CTCAE v. 4.0. Because many of the hematological AEs were attributed to underlying AML and began before study drug initiation, we did not include cytopenias in the safety analysis.

### 2.4. Endpoints

The primary outcome of this study was to assess efficacy by evaluating the complete remission (CR) and complete remission with incomplete hematologic recovery (CRi) rate (CR/CRi rate) after the second cycle of LD-AC+cladribine. Secondary outcomes included assessment of duration of remission (DOR), an overall response rate (ORR), OS, and safety. The objective response rate (ORR) was defined as the proportion of patients who achieved at least PR as their best overall response. DOR was defined as the time from achieving CR/CRi until the date of relapse or death from any cause. OS was defined as the time from AML diagnosis until the death due to any cause. Early death (ED) was defined as death from any reason within 30 and 56 days from the date of treatment initiation.

### 2.5. Statistical Analysis

The patients’ characteristics were summarized using frequency (percentage) for categorical variables and median (range) for continuous variables. OS and DOR were estimated by the Kaplan–Meier method. The log-rank test was used to compare OS between subgroups of patients. The Mann–Whitney U, Kruskall–Wallis, and chi-square tests were used to assess the effect of various variables on the CR/CRi rate. To determine independent factors associated with the CR/CRi rate, the logistic regression analyses were performed. The Cox proportional hazard model was used to estimate hazard ratios (HR) and 95% confidence intervals (95%CI). In the multivariate analysis of factors affecting OS, only clinically significant variables evaluated before treatment initiation were analyzed. All tests were two-sided and performed at a 0.05 significance level. Statistical analyses were performed using Statistica version 13.3 (StatSoft, Dell, Round Rock, TX, USA), Graph Pad Prism version 9.0 for Windows (GraphPad Software, San Diego, CA, USA), and SAS software (SAS Institute Inc., Cary, NC, USA).

## 3. Results

### 3.1. Patient Characteristics

From 1 February 2017 to 30 November 2019, a total of 117 elderly AML patients were enrolled. Overall, 117 (100%) and 82 (70%) patients received a first and second cycle of LD-AC+cladribine, respectively. The most common reasons for early discontinuation were death (*n* = 24; 20%) and withdrawal of consent (*n* = 13, 11%). Maintenance treatment with LD-AC was given to 78% (*n* = 49) of patients who achieved a response after two cycles of LD-AC+cladribine. The most common reasons for treatment discontinuation before the maintenance phase were death (*n* = 8; 7%) and withdrawal of consent (*n* = 5; 4%).

The median age in the study cohort was 70 years (range, 60–87) with 35% (*n* = 41) patients over the age of 75 years. The majority of patients had an ECOG PS ≥2 (60%, *n* = 70) and HCT-CI score ≥3 (58%, *n* = 67). Among 38 (32%) patients with sAML, 26 (22%) had AML related to myelodysplasia (AML-MRC), 7 (6%) had tAML, and 4 (3%) had history of prior myeloproliferative neoplasm (MPN). The median bone marrow blasts count was 51%. The baseline clinical characteristics is summarized in Table 1.

Cytogenetic data were available for 110 (94%) of patients. Intermediate and adverse cytogenetics were observed in the majority of patients (43.5% and 43.5%, respectively). In contrast, only 7% of patients had favorable cytogenetic risk. Molecular data were limited. Mutations in *FLT3* (both ITD and TKD) and *NPM1* genes were found in 13 (20%) and 11 (23%) patients out of 64 (55%) and 47 (40%) patients who were screened for these mutations, respectively. No mutations in other genes such as *CEBPA* and *RUNX1* were detected in 42 (36%) screened patients.

### 3.2. Efficacy

#### 3.2.1. Response

**Figure 1:**
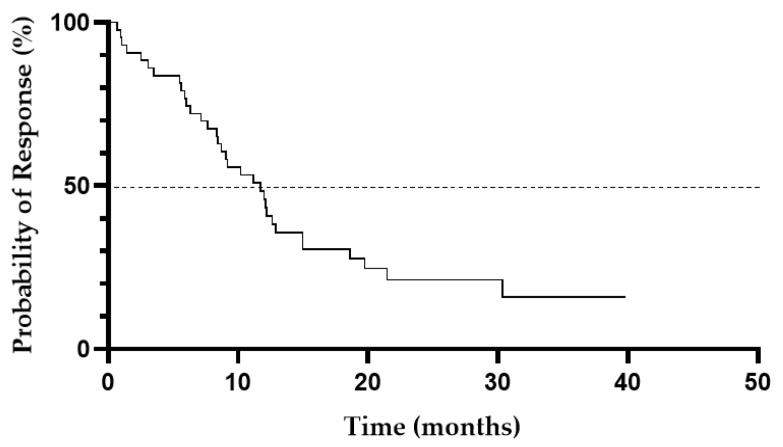
Duration of response in patients who achieved CR/CRi to LD-AC+cladribine.

The ORR was 54% (*n* = 63) with 37 (32%) CR, 6 (5%) CRi, and 20 (17%) PR. For patients aged ≥75 years, the CR/CRi and PR rate were 27% and 15%, respectively. A median time to CR and CRi achievement was 3 months (range, 1–6.4 months) and 2 months (range, 0.6–3.0 months), respectively. A median DOR was 11.7 months (95%CI, 8.5–15.0 months) for patients who achieved CR/CRi (Figure 1). Treatment response was sustained for more than 12 months in 34.8% (*n* = 15) of patients who achieved CR/CRi.

Both higher burden of comorbidities (defined as HCT-CI score ≥ 3) and adverse cytogenetic risk negatively affected the CR/CRi rate in the univariate analysis (CR/CRi rate 47.9% and 28.4%, respectively, for patients with HCT-CI score of 0–2 vs. ≥3, *p* = 0.03; CR/CRi rate 19.6% and 51%, respectively, for patients with adverse vs. intermediate cytogenetic risk, *p* = 0.009) (Table 2). However, in the multivariate analysis only adverse cytogenetic risk negatively affected the CR/CRi rate (Table 2).

Due to the small sample size, the reliable effect of somatic mutation status on achieving CR/CRi could not be investigated. CR/CRi was achieved in 5 out of 13 (38%) patients with *FLT3*-ITD or TKD and in 21 out of 51 (41%) patients with wild-type *FLT3* (*p* = 0.98). Furthermore, 5 of 11 patients (45%) with mutated *NPM1* and 15 of 36 (42%) patients with wild-type *NPM1* achieved CR/CRi (*p* = 0.82).

#### 3.2.2. Survival

**Figure 2:**
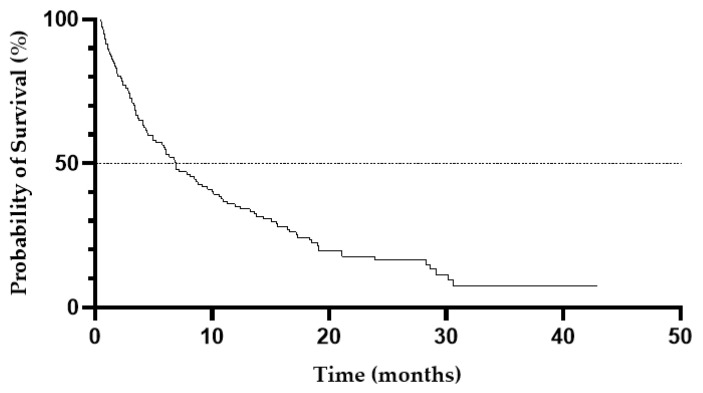
Overall survival for the entire study cohort.

**Figure 3:**
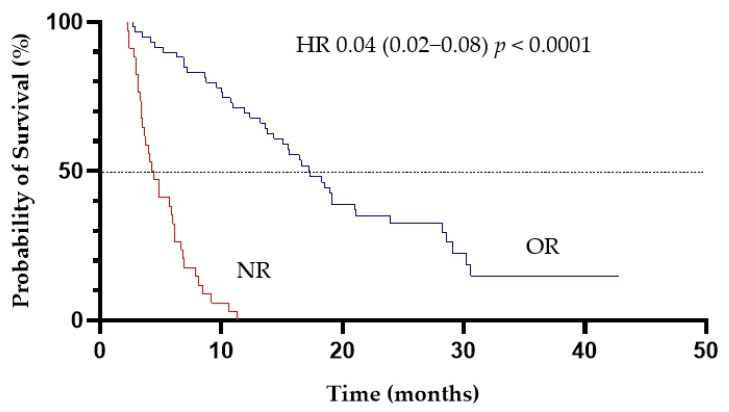
The effect of achieving an objective response (OR) to LD-AC+cladribine on overall survival (NR-no response).

**Figure 4:**
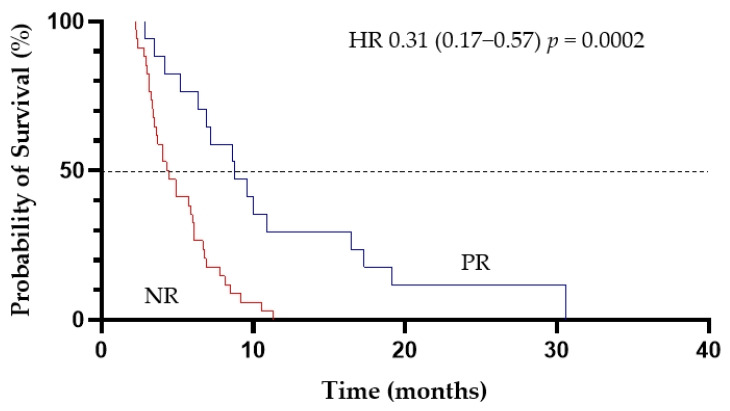
The effect of achieving partial response (PR) to LD-AC+cladribine on overall survival (NR-no response).

In total, 101 deaths were recorded during the study. The median OS for the entire cohort was 6.9 months (95%CI, 4.9–9.6 months) (Figure 2). Patients who achieved CR/CRi and PR had the median OS of 21 months (95%CI, 15.6–28.6 months) and of 8.8 months (95%CI, 5.2–16.4 months), respectively. There were significant differences in survival between responders and non-responders with the median OS of 17.3 months and of 4.4 months, respectively (HR, 0.04; 95%CI, 0.02–0.08; *p* < 0.0001) (Figure 3) (Table 3). Importantly, patients who achieved PR had improved survival compared with non-responders with the median OS of 8.8 months and of 4.4 months, respectively (HR, 0.31, 95%CI, 0.17–0.57; *p* = 0.0002) (Figure 4).

**Figure 5:**
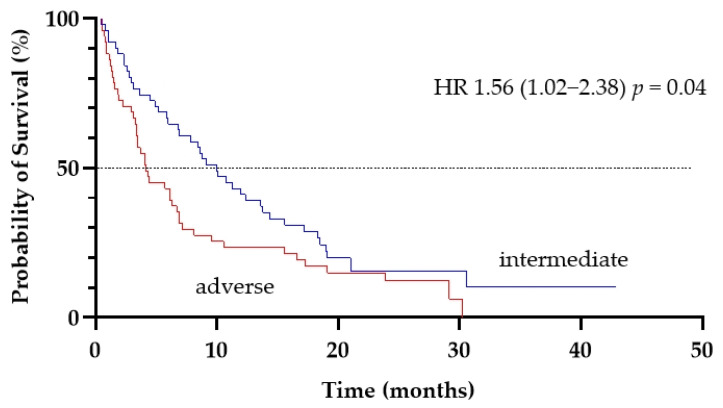
The effect of cytogenetic risk (adverse vs. intermediate) on overall survival of patients treated with LD-AC+cladribine.

The median OS for patients with adverse and intermediate cytogenetics were 4.2 months and 10 months, respectively (*p* = 0.04; Figure 5).

In the entire cohort, the estimated 6-month, 1-year, and 2-year OS rates were 55%, 35%, and 12.6%, respectively. Among responders, the estimated 1-year OS rate was 86.5%, 66.7%, and 25% for patients who achieved CR, CRi, and PR, respectively. Non-responders had a poor prognosis with the estimated 1-year OS rate of 0%.

Among the clinically significant factors assessed before the treatment initiation, HCT-CI score ≥3, adverse cytogenetic risk and age ≥75 years were associated with poor OS in univariate analysis. In the multivariate analysis, only age ≥75 years and adverse cytogenetic risk negatively affected OS (Table 3). Neither AML type (de novo AML vs. sAML) nor tumor burden before the treatment initiation (BM blasts of 20-30% vs. >30%, lactate dehydrogenase (LDH) serum activity below vs. above of 480 IU/L) had impact on OS.

Due to the small sample size, the reliable effect of somatic mutation status on survival could not be demonstrated. The median OS for patients with *FLT3*-ITD or TKD was 5.2 months (vs. 7.2 months for patients with wild-type *FLT3;* *p* = 0.8). Patients with mutated and wild-type *NPM1* had a median OS of 3.5 and 7.1 months, respectively (*p* = 0.75).

#### 3.2.3. Safety Profile

The early mortality (56-day) rate was 20.5% (*n* = 24), with 14 deaths occurring within 30 days of the treatment initiation. Seven (30%) and 15 (62%) ED were due to AML progression and infectious complications, respectively. The remaining 2 (8%) ED were a result of hemorrhagic and thrombotic complications. Overall, the toxicity profile of LD-AC+cladribine was predictable. All patients had at least one non-hematological AE. Infections were the most common non-hematological AEs and occurred in 57% of patients (*n* = 67). Grade 3 or 4 pneumonia, sepsis, neutropenic fever, and soft tissue infections were reported in 29 (25%), 17 (14.5%), 15 (13%), and 10 (8.5%) patients, respectively. Other grade 3 or 4 non-hematological AEs were observed in 34 (29%) patients with cardiac complications as the most common. The detailed characteristics of non-hematological grade 3 or 4 AEs are summarized in Table 4.

## 4. Discussion

The purpose of this study was to evaluate the efficacy and safety of the low-intensity regimen LD-AC+cladribine as a frontline treatment for elderly AML patients in poor PS or with severe comorbidities.

Our cohort well reflected the clinical heterogeneity of elderly AML patient population observed in real life. Approximately two-thirds of patients had either HCT-CI score ≥3 or ECOG ≥2 and one-third of patients were 75 years of age or older. The median OS for the entire cohort was 6.9 months and was significantly longer in patients who achieved CR/CRi (21 months) or PR (8.8 months) compared to those who did not respond to the therapy (4.4 months). Both advanced age (≥75 years) and high-risk cytogenetics were associated with an inferior OS.

Recently, Kadia et al. (Table 5) reported the results of the phase II study conducted by MD Anderson Cancer Center (MDACC study) evaluating LD-AC+cladribine alternating with decitabine in 118 patients aged 60 years or older with previously untreated AML or high-risk MDS [39]. The treatment plan consisted of 2 cycles (28 days each) of LD-AC+cladribine alternating with 2 cycles of decitabine for a total of 18 cycles. This combination regimen showed promising clinical activity with the CR/CRi rate of 68% and a median DOR of 14.7 months. The median OS for the entire cohort was 13.8 months and was significantly longer in patients who achieved a response (16.2 months) compared to non-responders (4.7 months). The regimen had an impressive safety profile with 4- and 8-week mortality rates of 1% and 7%, respectively [39]. Several significant differences between our and MDACC study should be highlighted. Firstly, we did not incorporate HMA in our treatment plan and patients in our study received two cycles of LD-AC+cladribine followed by LD-AC monotherapy given every 4 to 6 weeks until disease progression or unacceptable toxicity. Secondly, we included more patients aged 70 or older compared to MDACC study (57% vs. 44% of patients, respectively) and a significant proportion of our study population had poor PS (ECOG PS ≥3 in 20% of patients) and numerous comorbidities (HCT-CI score ≥3 in 58% of patients). In fact, every third patient in our study had both ECOG PS ≥2 and HCT-CI score ≥3. In contrast, only patients with adequate organ function and ECOG PS of 2 or less were enrolled to the MDACC study. Thirdly, nearly 90% of the patients in our study had an intermediate or unfavorable cytogenetic risk. Moreover, every fourth patient in our study had both adverse cytogenetics and poor PS (ECOG PS ≥2). In turn, in the MDACC study intermediate or adverse cytogenetics was observed in 21% and 41% of patients, respectively. These differences may explain a difference in CR/CRi rates and median OS for the entire cohorts between our and the MDACC study in favor of the latter. The long-term results of combinations of purine analogues (cladribine or clofarabine) with LD-AC administered alternately with decitabine in the frontline treatment of elderly unfit AML patients were recently evaluated by Kadia et al. [40]. Of the 248 patients included in the analysis, 41% were over 70 years of age (the median age was 69 years) and 44% had high-risk cytogenetics (patients with core-binding factor AML were excluded). The low-intensity regimens had significant anti-leukemic activity with CR/CRi rate of 66%. The median relapse-free survival and OS was 10.8 and 12.5 months, respectively [40]. These data suggest significant clinical benefits of combining low-dose chemotherapy with HMA and address the evolutionary nature of AML. As AML changes over time following different patterns of clonal evolution, alternating drugs with different mechanisms of action may play a key role in overcoming treatment resistance [41].

The main goal of AML treatment both in younger [43] and elderly or unfit [44] patients is to achieve a CR/CRi. Similar to Kadia et al., we show that patients who achieve CR or CRi during treatment have significantly improved survival compared to non-responders whose prognosis is poor. A significant finding of our study is that advanced age (≥75 years), even in patients with comorbidities and in poor PS, does not preclude the possibility of achieving CR when non-intensive chemotherapy is applied. In contrast to the prognostic value of CR, the significance of PR remains uncertain, and it is well known that long-term survival (beyond 3 years from diagnosis) without achieving CR is unlikely in AML patients [45]. However, it seems reasonable that for patients who fail to achieve CR, particularly those with severe comorbidities, PR may be of significant clinical value. This may be explained by reducing the burden of the leukemic clone, which leads to a decrease in transfusion dependency, relief of disease symptoms, and an improvement in quality of life. In support of this, we demonstrate here that unfit AML patients who achieve only PR during treatment with LD-AC+cladribine still have an improved outcome compared to those who fail to respond to applied therapy with the median OS of 8.8 and 4.4 months, respectively.

Our results should also be considered in the context of other approaches generally offered to unfit elderly AML patients, in particular, HMA. In an Italian observational study that enrolled 35 previously untreated AML patients not eligible for IC, treatment with azacitidine led to an ORR of 48% (CR, 23%; CRi, 8%; PR, 17%) and a median OS in the responders’ group of 13 months (Table 5). Importantly for comparison with our results, all patients with baseline leukocytosis above 10,000/μL did not respond to the treatment with azacitidine [22]. In a similar French study of 148 AML or high-risk MDS patients, response to azacitidine (at least PR) was observed in every third patient and an estimated OS at 1 and 2 years were 81% and 51% in responders’ group, respectively (Table 5) [21]. In turn, we show the estimated 1-year OS of 86.5%, 66.7%, 25%, in patients who achieved CR, CRi, and PR during treatment with LD-AC+cladribine, respectively. It should be emphasized that in our study patients had more comorbidities and worse PS than in the French study, where patients’ ineligibility for IC was determined based on age and some clinical and biological features of AML, while not on PS and comorbidities [21]. In a randomized phase III trial of elderly AML patients, frontline treatment with decitabine resulted with higher CR/CRi rate than LD-AC or BSC (25% and 10%, respectively). However, these results did not translate into significant differences in OS between the study groups (median OS of 7.7 and 5 months for decitabine and LD-AC or BSC group, respectively) [24]. In two phase II trials of elderly or unfit AML patients, the ORR for decitabine was 25–26% with the median OS of 5.5 to 7.7 months (Table 5) [25,26].

Treatment with LD-AC+cladribine was associated with 30- and 56-day mortality rates of 12% and 20%, respectively, which were higher than reported in the MDACC study (4- and 8-week mortality rates of 1% and 7%, respectively [39]), but similar to those noted for azacitidine (4-week mortality rate of 10% in the Italian observational study [22] and 30- and 60-day mortality rates of 6.6% and 16.2%, respectively in the phase III trial [20]), decitabine (30- and 60-day mortality rates of 7% [26] and 19.7%, respectively [24]), and LD-AC (60-day mortality rate of 23% [24]).

The toxicity profile of LD-AC+cladribine was predictable. In line with other studies in this difficult-to-treat AML patient population [24,25,39,46] infectious complications were most commonly reported non-hematological toxicity with pneumonia, sepsis, and neutropenic fever complicating treatment in 25%, 14.5%, and 13% of cases, respectively.

It was shown that the burden of comorbidities is predictive for ED and survival in AML patients over 60 years of age receiving intensive induction therapy with ED rates of 3%, 11%, and 29% and the median OS of 45, 31, and 19 weeks in patients with HCT-CI score of 0, 1–2, and 3 or more, respectively [14]. In contrast, in our study high-risk HCT-CI showed no statistical significance in multivariate analysis for OS, indicating that even patients with a high burden of comorbidities may benefit from treatment with LD-AC+cladribine.

With the advent of novel highly active agents, especially venetoclax [42,46,47], glasdegib [48] and IDH1/2 inhibitors [49,50], the role of chemotherapy as a frontline treatment of elderly and unfit AML patients is questioned [51]. Recently, the phase III VIALE-A trial confirmed the superiority of venetoclax, combined with azacitidine over azacitidine alone in the frontline therapy for elderly (over 75 years of age) and/or unfit AML patients (Table 5) [42]. Treatment with venetoclax and azacitidine was associated with significant improvement in CR/CRi rate (66% vs. 28%) and OS (median 14.7 vs. 9.6 months) compared to the control arm. There were no significant differences in the 30-day mortality rates between the study cohorts (7% vs. 6%, in active and control arm, respectively) [42]. The clinical benefits of combining venetoclax with decitabine [46] or LD-AC [47] in the first-line treatment of AML patients not eligible for IC have also been demonstrated. In this context, the incorporation of venetoclax and azacitidine into the LD-AC+cladribine regimen to further improve outcomes seems to be a reasonable option and preliminary results of the phase II study indicate both an acceptable safety profile and promising clinical activity of this combo as frontline therapy for AML patients unfit for IC [52]. It has been suggested that particular benefits of this drug combination may be achieved by patients with adverse cytogenetics, for whom, as our results indicate, LD-AC+cladribine alone is not an effective therapeutic option.

Several limitations of our study need to be underlined. Firstly, it was an observational study without a comparator arm. Secondly, there were only a few patients with favorable cytogenetics; therefore, the impact of cytogenetic risk on treatment outcomes could not be fully demonstrated. Thirdly, we suffered from a lack of molecular data; hence the impact of recurrent genetic mutations on the prognosis of patients treated with LD-AC+ cladribine could not be evaluated.

## 5. Conclusions

In summary, our study shows that the low-intensity treatment with LD-AC+cladribine is a beneficial therapeutic option with an acceptable safety profile in the difficult-to-treat population of elderly AML patients not eligible for IC.

## Figures and Tables

**Table 1:** Patient and disease baseline characteristics (*n* = 117).

**Parameter**	**Value**
Age (years)	
Median (range)	70 (60–87)
>70, *n* (%)	67 (57)
≥75, *n* (%)	41 (35)
ECOG performance status, *n* (%)	
0	11 (9.5)
1	36 (31)
2	47 (40)
3	19 (16)
4	4 (3.5)
HCT-CI score, *n* (%)	
Median (range)	3 (0–10)
0–2	48 (42)
≥3	67 (58)
Sex, *n* (%)	
Female	66 (56)
Male	51 (44)
Bone marrow blasts count, *n* (%)	
20–29%	15 (13)
30–50%	41 (35)
≥50%	61 (52)
LDH serum activity >480 IU/L, *n* (%)	51 (46)
Serum creatinine >1.3 mg/dL, *n* (%)	23 (20)
Cytogenetic risk, *n* (%)	
Favorable	8 (7)
Intermediate	51 (43.5)
Adverse	51 (43.5)
Lack of metaphases	7 (6)
AML status, *n* (%)	
De novo	79 (67.5)
Secondary	38 (32.5)
Somatic mutation, *n* (%)	
*FLT3*-ITD or TKD ^(1)^ *NPM1*^(2)^	13 (11) 11 (9)
Coexistence of somatic mutations, *n* (%)	
*FLT3*-ITD and TKD (−), *NPM1* (−)	31 (26)
*FLT3*-ITD and TKD (−), *NPM1* (+)	4 (3)
*FLT3*-ITD or TKD (+), *NPM1* (−)	4 (3)
*FLT3*-ITD or TKD (+), *NPM1* (+)	6 (5)
Treatment for antecedent hematologic disorders, *n* (%)	
Azacitidine	7 (6)
Hydroxyurea	3 (3)
LD-AC and azacitidine	1 (1)
not specified	1 (1)

^(1)^ Data were available for 64 (55%) patients; ^(2)^ data were available for 47 (40%) patients. Abbreviations: AML, acute myeloid leukemia; ECOG, the Eastern Cooperative Oncology Group; HCT-CI, the hematopoietic cell transplantation comorbidity index; ITD, internal tandem duplication; LD-AC, low-dose cytarabine; LDH, lactate dehydrogenase; TKD, tyrosine kinase domain.

**Table 2:** Univariate and multivariate analyses of factors affecting the CR/CRi rate.

Independent Variable	Univariate Analysis		
	CR/CRi	Non-CR	*p*
	*n* (%)	*n* (%)	
Age			
60–74 y	32 (42%)	44 (58%)	0.1
≥75 y	11 (26.8%)	30 (73.2%)	
ECOG performance status			
0–1	16 (34%)	31 (66%)	0.67
≥2	27 (38.6%)	43 (61.5%)	
HCT-CI score			
0–2	23 (47.9%)	25 (52.1%)	0.03
≥3	19 (28.4%)	48 (71.6%)	
Cytogenetic risk			
Intermediate	26 (51%)	25 (49%)	0.009
Adverse	10 (19.6%)	41 (80.4%)	
Bone marrow blasts count			
20–30%	5 (33.3%)	10 (66.7%)	0.11
>30%	38 (37.3%)	64 (62.7%)	
AML status			
De novo	33 (41.7%)	46 (58.3%)	0.15
Secondary	10 (26.3%)	28 (73.6%)	
Serum LDH activity			
<480 IU/L	21 (34.4%)	40 (65.6%)	0.6
≥480 IU/L	20 (39.2%)	31 (60.8%)	
Serum creatinine			
<1.3 mg/dL	35 (37.2%)	59 (62.8%)	0.83
≥1.3 mg/dL	8 (34.7%)	15 (65.3%)	
**Variable**	**Multivariate analysis**		
	**Odds ratio for CR/CRi rate (95%CI)**		***p***
Age ≥75 years	0.39 (0.14–1.09)		0.072
Adverse cytogenetics (vs. intermediate cytogenetics)	0.28 (0.11–0.71)		0.007

Abbreviations: AML, acute myeloid leukemia; CR, complete response; CRi, complete response with incomplete hematologic recovery; ECOG, the Eastern Cooperative Oncology Group; HCT-CI, the hematopoietic cell transplantation comorbidity index; LDH, lactate dehydrogenase.

**Table 3:** Baseline factors affecting overall survival in univariate and multivariate.

Title	Univariate Analysis		Multivariate Analysis	
Variable	HR for Death (95%CI)	*p*	HR for Death (95%CI)	*p*
Age (≥75)	1.51 (1.01–2.27)	0.047	1.66 (1.05–2.62)	0.032
HCT-CI score (≥3)	1.61 (1.07–2.41)	0.023	1.61 (0.98–2.61)	0.054
Cytogenetic risk (adverse)	1.55 (1.02–2.36)	0.042	1.67 (1.05–2.67)	0.031
ECOG PS (>1)	0.99 (0.67–1.47)	0.971	-	-

Abbreviations: CI, confidence interval; ECOG, the Eastern Cooperative Oncology Group; HCT-CI, the hematopoietic cell transplantation comorbidity index; HR, hazard ratio; PS, performance status.

**Table 4:** Grade 3 or 4 non-hematological adverse events at least possibly related to the study treatment.

Grade 3 or 4 Infectious Adverse Events		Grade 3 or 4 Non-Infectious Adverse Events	
Grade 3 or 4 Infectious Adverse Events	*n* (%)	Grade 3 or 4 Non-Infectious Adverse Events	*n* (%)
Pneumonia	29 (25%)	Cardiac arrhythmia	5 (4%)
Sepsis	17 (14.5%)	Cardiac, other (CHF, ACS)	3 (2.5%)
Neutropenic fever	15 (13%)	Constipation	6 (5%)
Soft tissue infection	10 (8.5%)	Pruritis	2 (1.7%)
Clostridioides difficile infection	3 (2.5%)	Thrombosis or pulmonary embolism	2 (1.7%)
Other	10 (8.5%)	Acute kidney injury	2 (1.7%)
		Other	14 (12%)

Abbreviations: CHF, congestive heart failure; ACS, acute coronary syndrome.

**Table 5:** Selected studies evaluating frontline treatment for unfit elderly AML patients.

Studied Population	*n*	Main Inclusion Criteria	Median (Range) Age (Years) of the Study Participants	Selected AML Characteristics	Treatment	Outcomes	References
Previously untreated AML or HR-MDS	118	Age ≥60 years; ECOG PS ≤2; adequate organ function	69 (49–85); patients aged ≥70 years, 44%	Cytogenetics: HR, 41%; IR, 21% tAML, 17%	LD-AC+cladribine for 2 cycles alternating with decitabine for 2 cycles, for up to 18 cycles	CR/CRi rate, 68%; median DOR, 14.7 months; median OS, 13.8 months	[39]
Previously untreated AML	35	Age ≥18 years; adequate renal and hepatic function; the absence of alternative therapeutic options	77 (46–87); patients aged ≥70 years, 91%	Cytogenetics: HR, 23%; IR, 37% tAML, 3%	Azacitidine	CR/CRi rate, 31%; median DOR, 6 months; median OS, 9 months	[22]
Previously untreated AML	149	Ineligibility for IC due to age and/or HR AML characteristics, including HR cytogenetics or post-MDS AML or post-MPN AML or tAML (ECOG PS <2 in 71% of participants)	74 (31–91); patients aged ≥65 years, 83%	Cytogenetics: HR, 40%; IR, 53% tAML, 20%	Azacitidine	CR/CRi rate, 23%; median DOR, not provided; median OS, 9.4 months	[21]
Previously untreated AML	55	Age ≥60 years and HR or IR cytogenetics; ECOG PS ≤2; adequate renal and hepatic function	74 (61–87); patients aged ≥70 years, 62%	Cytogenetics: HR, 45%; IR, 53% tAML, 7%	Decitabine	CR/CRi rate, 26%; median DOR, not provided; median OS, 7.7 months	[26]
Previously untreated AML	Azacitidine + venetoclax arm: 286 Azacitidine + placebo arm: 145	Age ≥75 years or the presence of specific clinical conditions precluding IC; ECOG PS 0–2 for patients ≥75 years of age; ECOG PS 0–3 for patients ≥18 to 74 years of age; adequate renal and hepatic function	Azacitidine + venetoclax arm: 76 (49–91); patients aged ≥75 years, 61% Azacitidine + placebo arm: 76 (60–90); patients aged ≥75 years, 60%	Azacitidine + venetoclax arm: HR cytogenetics, 36%; IR cytogenetics, 64% Azacitidine + placebo arm: HR cytogenetics, 39%; IR cytogenetics, 61%	Azacitidine + venetoclax versus azacitidine + placebo	Azacitidine + venetoclax arm: CR/CRi rate, 66.4%; median DOR, 17.5 months; median OS, 14.7 months Azacitidine + placebo arm: CR/CRi rate, 28.3%; median DOR, 13.4 months; median OS, 9.6 months	[42]

Abbreviations: AML, acute myeloid leukemia; CR, complete response; CRi, complete response with incomplete hematologic recovery; DOR, duration of response; ECOG, the Eastern Cooperative Oncology Group; HR, high risk; IC, intensive chemotherapy; IR, intermediate risk; LD-AC, low-dose cytarabine; MDS, myelodysplastic syndrome; MPN, myeloproliferative neoplasm; OS, overall survival; PS, performance status.

## Data Availability

The data presented in this study are available on request from the corresponding author.
